# Retraction: Magnetic Fe_3_O_4_@NiO hierarchical structures: preparation and their excellent As(v) and Cr(vi) removal capabilities

**DOI:** 10.1039/d1ra90145a

**Published:** 2021-09-15

**Authors:** Shouwei Zhang, Jiaxing Li, Tao Wen, Jinzhang Xu, Xiangke Wang

**Affiliations:** School of Material Science and Engineering, Hefei University of Technology Hefei 230031 P. R. China; Key Laboratory of Novel Thin Film Solar Cells, Institute of Plasma Physics, Chinese Academy of Sciences P.O. Box 1126 Hefei 230031 P. R. China xkwang@ipp.ac.cn +86-5515591310 +86-551-5592788

## Abstract

Retraction of ‘Magnetic Fe_3_O_4_@NiO hierarchical structures: preparation and their excellent As(v) and Cr(vi) removal capabilities’ by Shouwei Zhang *et al.*, *RSC Adv.*, 2013, **3**, 2754–2764, DOI: 10.1039/C2RA22495J.

The Royal Society of Chemistry, with the agreement of the named authors, hereby wholly retracts this *RSC Advances* article due to concerns with the reliability of the data in the published article. The authors requested to retract this article because they admitted that the TEM characterization of the Fe_3_O_4_@NiO hierarchical microspheres in Fig. 4c was duplicated from the characterization of Fe_3_O_4_@NiAl-LDH microspheres in Fig. S4B from a *J. Am. Chem. Soc.* paper by Mingfei Shao *et al.* without permission.^1^ The authors would like to apologise to the authors of ref. 1, and for any inconvenience to readers.

Signed: Shouwei Zhang, Jiaxing Li, Jinzhang Xu and Xiangke Wang

Date: 11th August 2021

Tao Wen was contacted but did not respond

Retraction endorsed by Laura Fisher, Executive Editor, *RSC Advances*

## Supplementary Material
